# Digestive stability and transport ability changes of β-lactoglobulin–catechin complexes by M cell model *in vitro*

**DOI:** 10.3389/fnut.2022.955135

**Published:** 2022-08-22

**Authors:** Yan Dai, Ruoting Yang, Yuting Yan, Yong Wu, Xuanyi Meng, Anshu Yang, Zhihua Wu, Linbo Shi, Xin Li, Hongbing Chen

**Affiliations:** ^1^State Key Laboratory of Food Science and Technology, Nanchang University, Nanchang, China; ^2^School of Food Science and Technology, Nanchang University, Nanchang, China; ^3^Sino-German Joint Research Institute, Nanchang University, Nanchang, China; ^4^Jiangxi Province Key Laboratory of Food Allergy, Nanchang University, Nanchang, China; ^5^Department of Pathogen Biology and Immunology, School of Basic Medical Sciences, Nanchang University, Nanchang, China

**Keywords:** β-lactoglobulin, catechins, free radical crosslinking, digestive ability, crosslinking mechanism, transport capacity

## Abstract

The current research on interaction between catechin and protein has focused on non-covalent crosslinking, however, the mechanism of free radical-induced crosslinking between catechin and β-lactoglobulin (BLG) is not known. In this study, BLG bound to four catechins [epicatechin (EC), epigallocatechin (EGC), epicatechin gallate (ECG), and epigallocatechin gallate (EGCG)]. The structure change of complex was investigated by circular dichroism spectroscopy, ultraviolet-visible (UV-vis) spectroscopy and Acid and 8-Anilino-1-naphthalenesulfonic acid (ANS) fluorescence spectroscopy. M cell model was constructed to evaluate the transintestinal epithelial transport capacity of complex digestive products. The results showed that catechins were covalently bound to BLG by C-S and C-N bonds and their binding content was EGCG>EGC>ECG>EC. Moreover, catechins could change the secondary structure of BLG, with the decrease of α-helix and reduction of the irregular coilings, which leads to the loose spatial structure of the protein. Moreover, the catechin could enhance further the digestibility of BLG. Transport capacity of digestive products of M cell model was about twice of that of the Caco-2 cell model, indicating that M cell model had better antigen transport capacity. The difference between groups indicated that the transport efficiency of digestive products was decreased with the presence of catechin, in which BLG-EGCG and BLG-EGC groups were transported more strong than those of BLG-EC and BLG-ECG groups. The transport efficiency of BLG-catechin complexes were lower than that of BLG, indicating that catechin had the protective and repair roles on intestinal barrier permeability.

## Introduction

Plant polyphenol is a kind of secondary metabolites with polyphenol structure which exists widely in plants, which showed biophysical function in antioxidant, anti-inflammatory, reduce bacteriostatic and cardiovascular risk (1). It is frequently used as additives and functional ingredients in the food industry. Tea is a good source of plant polyphenols. Catechins are the main components of polyphenols in tea, which accounted for more than 10% of the polyphenols in dried tea (2). Catechins are mainly composed of epicatechin (EC), epigallocatechin (EGC), epicatechin gallate (ECG), and epigallocatechin gallate (EGCG) (3). Catechins have become one of the most popular antioxidants because of their functional characteristics, since catechins have been found to have the effects of reducing obesity, lowering blood lipids and inhibiting the growth of cancer cells due to their special active substances. When polyphenols coexisted with proteins covalently or non-covalently, it could change the structure and properties of proteins, thus affecting the sensory, functional and nutritional properties of foods (1, 4). Studies have shown that catechins can improve foaming ability and synergistic scavenging of free radicals for β-lactoglobulin (BLG) (5, 6), with the secondary and space structural changes (7) and modification of surface hydrophobicity of proteins (8).

β-lactoglobulin is one of the most important proteins in milk (9), it is a water-soluble protein and usually to be used as food additives for its nutritional and functional activities (10). Further, after BLG is digested in the gastrointestinal tract, it will degrade into peptides with lower molecular weight, which then cross the intestinal barrier and elicit an immune response. Static *in vitro* simulation of gastrointestinal digestion has been widely used in the study of food digestion with the advantages of simple operation, termination at any time and easy adjustment. Digestion and absorption of digestive products through the intestinal barrier is an important link for substances to exert their nutritional value and is also a pre-requisite for human immune response (11). Structurally speaking, it has been reported that BLG monomer contains multiple holes that can bind hydrophobic ligands (12). Therefore, it is often used as a ligand for some hydrophobic small molecules like phenols. Reports of non-covalent crosslinking between BLG and polyphenols has been studied (13, 14), the interactions may cause of structure and stability changes of BLG (15). It has been found that the interaction between polyphenols (16) and milk protein could increase the sensitivity of protein to pepsin digestion and combination of phenols and proteins like BLG may be a potential solution to change the physical and chemical properties of protein.

However, the non-covalent crosslinking products of polyphenols and proteins were extremely unstable (17). Therefore, it is of great significance to investigate the stable covalent crosslinking products of catechins and BLG, and its corresponding physic al and chemical properties, digested characteristics for study of milk proteins. In this study, we explored the conjugation of catechins in tea with bovine BLG *via* covalent crosslinking. Change of structural, molecular weight and reactive groups was analyzed. Digestion models *in vitro* was constructed to assess the digestive stability and the digestive products’ transportation was evaluated by two cell model.

## Materials and methods

### Materials

β-lactoglobulin, 5,5′-dithio-bis-(2-nitrobenzoic acid), pepsin, pancreatin, Calcium chloride anhydrous, Sodium Glycodeoxycholate, and Sodium Taurocholate were obtained from Sigma-Aldrich (St. Louis, MO, United States). EC, ECG, EGC, and EGCG were purchased from Mackin (Shanghai, China). Dulbecco’s Modified Eagle Medium (DEME), Roswell Park Memorial Institute (RPMI) 1640 medium, D-hanks were supplied by Solarbio (Beijing, China). All other reagents are analytically grade or higher grade.

### Sample preparation

The free radical method was performed as described (18) with a minor modification. 5 mg/mL BLG solution was prepared with distilled water and magnetically stirred for 1 h at 25°C to form aqueous phase. Then, 0.5 mL 30% H_2_O_2_ and 0.25 g ascorbic acid were added into each 100 mL protein solution and the mixture was placed at 25°C for 2 h. Subsequently, 50 mg catechins (EC, ECG, EGC, and EGCG) were added to each 100 mL protein solution at 25°C and exposed in the air for 24 h. Finally, the unbound catechins and salt ions were intercepted by dialysis with 3, 400 Da cutoffs for 48 h at 4°C.

### Characterization of β-lactoglobulin–Polyphenol covalent conjugates

#### Sodium dodecyl sulphate-polyacrylamide gel electrophoresis

Sodium dodecyl sulphate-polyacrylamide gel electrophoresis (SDS-PAGE) was performed as usual. In a nutshell, the sample (1 mg/mL) was heated at 100°C for 5 min to denature and then mixed with the loading buffer. The bands of protein were visualized with G:BOX F3 Gel Documentation System (Syngene, Cambridge, United Kingdom).

#### Determination of free amino groups

We referred to Rawel’s experimental scheme (19), that OPA method was used to determine the content of free amino group in BLG and BLG-catechin complexes.

#### Determination of sulfhydryl thiol groups

We detected the content of sulfhydryl groups in BLG and BLG-catechin complexes by using Ellman method as described (20). Firstly, Ellman’s regents were configured: 8 mg DTNB [5,5′-dithio-bis-(2-nitrobenzoic acid)] was dissolved in 2 mL Tris-glycine buffer containing 0.086 M Tris, 0.09 M glycine, and 4 mM EDTA. Subsequently, 50 μl Ellman’s reagents were added into 3 mg/mL samples and mix well. Finally, the mixture was maintained at 25°C for 1 h. And the absorbance value was measured at 412 nm. The content of the sulfhydryl was calculated as follows:


$$\mathrm{Sulfhydryl}\mathrm{thiol}\left(\text{SH}\right)\;(\frac{\mu mol}{\text{mg}})=73.53\times {\text{A}}_{412}/\text{C}$$

[C represents the concentration of sample protein (mg/mL)].

### Structural analysis of β-lactoglobulin-catechin complexes

#### Circular dichroism spectra

To detect the secondary structure of BLG and BLG-catechin complexes, circular dichroism (CD) spectroscopy was performed using a Pistar π-180 spectroscopy polarimeter (Applied Photophysics Ltd., London, United Kingdom). The scanning spectra was ranging from 190 to 240 nm at a rate of 100 nm/min and the bandwidth was 2 nm. The contents of secondary structure were calculated by using online software.^1^

#### Measurement of ultraviolet-visible absorbance

UV absorption spectral analysis was performed using a TU-1901 UV spectrophotometer (Puxi, Beijing, China). The sample was diluted into a concentration of 0.2 mg/mL, and the protein spatial structure of BLG-catechin complexes were determined at the wavelength ranging from 250 to 350 nm.

### Simulated gastrointestinal tract model *in vitro*

#### Simulation of *in vitro* digestion

β-lactoglobulin and BLG-catechin complexes were digested using pepsin according to the United States Pharmacopeia. A reaction system with a volume of 10 mL was designed, the centration of protein was 3 mg mL^–1^, and the ratio of protein to pepsin (20,000 U mL^–1^) was 25:4 (v/v). Before adding pepsin, protein and pepsin were pre-incubated at 37°C for 10 min. Aliquots were obtained at timed intervals. Subsequently, the digestion reactions were terminated at pH 7.0 by adding 1 M Na_2_CO_3_. Samples were frozen and stored at −20°C until use.

A 5 mL of the digestible product after gastric digestion was taken out, 1.592 mL of intestinal storage solution, 2 mL of 0.05 M bile salt, 20 μL of 0.3 M CaCl_2_ and 2 mL of trypsin (9 U/mg protein) were added, the pH was adjusted to 6.8, and distilled water was added to make the total system 10 mL. Aliquots were obtained at timed intervals. And the enzyme was inactivated in boiling water for 5 min.

#### Tricine-sodium dodecyl sulphate-polyacrylamide gel electrophoresis

Tricine-SDS-PAGE was conducted according to the technique reported by Schägger to separate proteins within the range of 1–100 kDa. The gel was prepared by overlaying a polymerized separating gel (16.5 and 10%) directly with a 4% sample (stacking) gel; the protein samples were mixed with the loading buffer at a ratio of 1:4 under non-reducing conditions. Electrophoresis was performed at a constant voltage of 30 V in the 4% sample (stacking) gel for 1 h and 100 V in the separating gel for 2.5 h. The image was performed using the GS-800 gel imaging system.

### Construction and evaluation of M cell model *in vitro*

#### Culture and construction of Caco-2 cells and Raji B-cells

The culture of Caco-2 cells and Raji B-cells were referred to MC Ponce de León-Rodríguez et al. (21). The cells were rigorously cultured aseptically. After the Caco-2 monolayer cell model was constructed, and the Transwell plate was cultured for 15 days. The fluid on the BL side of the Transwell plate was sucked out, and 1 × 10^6^ cell/ well Raji B-cells were added. The fluid on the AP side was changed daily, and the upper fluid on the BL side was absorbed daily, and half amount of the fluid was changed. In order to verify whether M cells were differentiated, resistance values were detected on the Caco-2 monolayers grown after 15 days and the Transwell plate with Raji B-cells added. Resistance values were recorded on days 10, 12, 14, 16, 17, 18, 19, 20, and 21.

#### Evaluation of cell proliferation toxicity

The cells were diluted to 2 × 10^5^ cell/mL with complete medium at 100 μL/well. In the toxicity test, the controls and the samples were set. It can be used for toxicity test when the degree of cell fusion is above 85%. For the control groups, 90 μL incomplete medium and 10 μL sterile PBS were added to each well, for the sample groups, 90 μL incomplete medium and 10 μL sterile end products (25, 50, 75, and 100 μg/mL) were added to each well. The liquid in the well was discarded and added with 100 μL complete medium and 10 μL CCK-8 reagent. The culture was continued for 2 h and the absorbance was measured at 450 nm.

#### Transport analysis of digestive products in M cell model

After the Transwell plate was tested for membrane permeability, the Transwell plate was taken out of the incubator, left at room temperature for 5 min, the AP side and BL side liquid were discarded, and Hank’s solution was carefully added to moisten and wash twice. Hank’s solution was added again and placed in the cell incubator for 30 min. Hank’s solution in the well was discarded, 0.5 mL of sterile digestion sample was added to the AP side, 1.5 mL of Hank’s solution was added to the BL side, and the solution was cultured in the incubator for 4 h. Draw fluid from AP side and BL side. BCA kit was used to detect the change of peptide concentration and analyze the transport capacity of the sample.

### Statistical analysis

All data were processed in GraphPad 8.0 software and reported as the mean ± standard deviation (SD).

## Results

### Molecular weight change of β-lactoglobulin-catechin complexes

**FIGURE 1:**
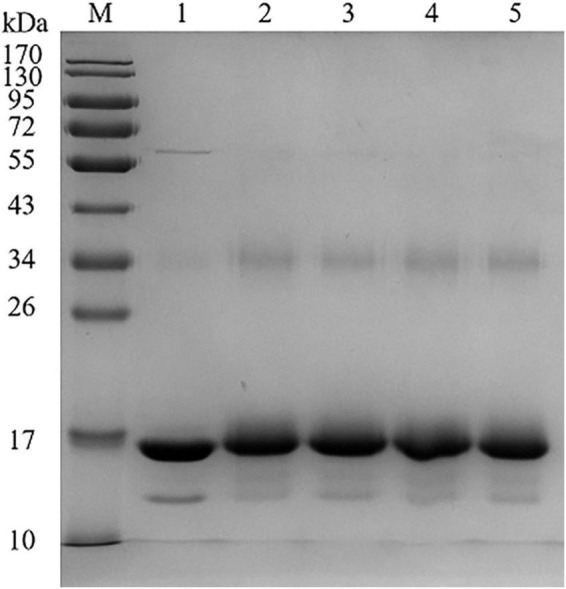
Sodium dodecyl sulphate-polyacrylamide gel electrophoresis (SDS-PAGE) of BLG-catechin complexes (M: Marker; Lane 1: BLG; Lane 2: BLG-EC; Lane 3: BLG-ECG; Lane 4: BLG-EGC; Lane 5: BLG-EGCG).

Different catechins binding to BLG was identified by SDS-PAGE, as shown in Figure 1. According to the electrophoresis diagram, the main band of BLG was located at about 17 kDa, and the band of conjugated BLG slightly shifted up, indicating the formation of BLG-catechin complexes. Since the bonds between catechins and BLG were not destroyed by SDS or mercaptoethanol, it indicates that the complex was formed by covalent bonds. This is also consistent with the study by Ali et al. (22) about the mechanism of the formation of covalent bonds between polyphenol oxidase and BLG.

### Analysis of total phenol binding equivalent

In order to determine the contents of different phenols bound to BLG, the Folin-phenol method was performed. We could find from Table 1, the contents of different catechins in complexes were different, among which most ratio of EGCG bound to BLG, while the least was EC bound. Compared with EC, EGCG had a gallic acid substituent at the third position of the C-ring, and the gallic acid group may promote the binding ability of catechins to BLG. Similarly, the binding ability of BLG to ECG was much higher than that of BLG-EC. The catechin content in BLG-EGC covalent complex was only slightly lower than BLG-EGCG, which might be due to the fact that EC and ECG were dihydroxy catechins, while EGC and EGCG are trihydroxy catechins. The difference in the number of hydroxyl groups was also a major factor affecting the covalent binding of proteins (23).

**TABLE 1:** Total phenolic content of BLG and BLG-catechin complexes.

Sample	Total phenol binding equivalent (mg/mL)
BLG-control	–
BLG-EC	28.710 ± 0.41^d^
BLG-ECG	42.075 ± 0.92^c^
BLG-EGC	42.372 ± 0.53^b^
BLG-EGCG	45.155 ± 1.37^a^

Different superscript letters denote significant differences (*p* < 0.05).

### Analysis of reactive groups

In our study, we detect the contents of free amino group, sulfhydryl group and tryptophan group between BLG–catechin complexes. Table 2 showed the contents of free amino groups of complexes decreased significantly. Among them, the content of free amino group was mainly from the side chain of lysine (24). The results showed that the catechins binding to BLG was through the lysine residues of the side chain of protein. The content of sulfhydryl groups was determined by 8 M urea, which could inhibit the conversion of free sulfhydryl groups to disulfide bonds. The amount of sulfhydryl groups in the BLG-catechin complexes was far less than that in the untreated BLG, indicating that the C-S bond were formed between the amino acid residues of BLG and catechin. In the natural state of BLG, tryptophan was wrapped inside the hydrophobic group, but the tryptophan content of BLG was significantly increased after the combination of catechin and BLG, which suggested that the complex structure has changed and lead to the tryptophan expose to the molecular surface.

**TABLE 2:** Total free amino, total sulfhydryl groups, tryptophan and tyrosine groups content of BLG-catechin complexes.

Sample	The content of free amino (nmol/mg)	The content of total sulfhydryl groups (nmol/mg)	The content of total tryptophan groups (ng/mg)	The content of tyrosine groups (ng/mg)
BLG-control	743.31 ± 6.6^a^	29.83 ± 0.26^a^	131.84 ± 11.78^de^	438.47 ± 9.79^a^
BLG-EC	657.31 ± 6.62^cde^	21.53 ± 0.09*^b^*	231.14 ± 19.09^c^	409.37 ± 20.84^de^
BLG-ECG	715.05 ± 4.41^b^	20.72 ± 0.06^c^	469.41 ± 19.19^a^	400.11 ± 14.35*^e^*
BLG-EGC	648.88 ± 2.21^e^	18.15 ± 0.2^d^	117.38 ± 11.17^e^	426.57 ± 12.39^cde^
BLG-EGCG	649.51 ± 11.03^de^	14.4 ± 0.09^e^	240.93 ± 17.00^bc^	436.49 ± 15.50^bcd^

Different superscript letters denote significant differences (*p* < 0.05).

### Analysis of structural changes of β-lactoglobulin–Catechins complexes

**FIGURE 2:**
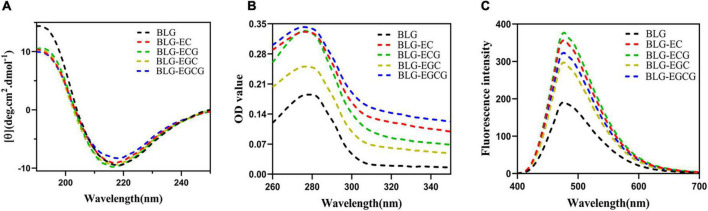
Spectrum change of BLG after binding to catechin panel **(A)** represented the Far-UV circular dichroic spectra of BLG-catechin complexes; panel **(B)** represented UV-Vis spectra of BLG-catechin complexes; panel **(C)** represented Acid and 8-Anilino-1-naphthalenesulfonic acid (ANS) fluorescence spectra of BLG-catechin complexes.

As shown in Figure 2, the absolute θ-values of BLG-catechin complexes at 208 nm were all reduced, indicating that the content of α-helical content of the catechin-related product was reduced. Table 3 displayed the content of α-helix, β-sheet and random coiled structure of BLG after crosslinking with catechin.

**TABLE 3:** Secondary structure contents of BLG-catechin complexes.

Sample	α -Helix	β -Sheet	β -Turns	Unordered
BLG	24.5	29.5	20.5	25.5
BLG-EC	19.9	29.7	21.9	28.4
BLG-ECG	22.0	28.8	21.5	27.8
BLG-EGC	21.2	28.0	22.3	28.4
BLG-EGCG	20.5	29.1	22.0	28.3

As shown in Figure 2B, the UV absorption of BLG-catechin complexes were significantly increased, and the descending order of UV absorption intensities was BLG-EGCG, BLG-ECG, BLG-EC, BLG-EGC, and BLG. These results indicated that the spatial structure of BLG after cross-linking with catechins was expanded and catechins could lead to the exposure of tryptophan, tyrosine and phenylalanine groups in protein molecules (25).

Acid and 8-Anilino-1-naphthalenesulfonic acid (ANS) was widely used fluorescent “hydrophobic probe” that it is widely used to monitor changes of protein hydrophobicity (26). As a structural property of proteins, surface hydrophobicity also had a great influence on the functional properties of proteins. It was shown in Figure 2C that the surface hydrophobicity of covalent complexes was enhanced, since it exposed more hydrophobic amino acids, thus loosening the spatial structure of the protein and leading to the enhancement of the surface hydrophobicity of BLG.

### Analysis of changes in digestion stability of β-lactoglobulin-catechin complexes

**FIGURE 3:**
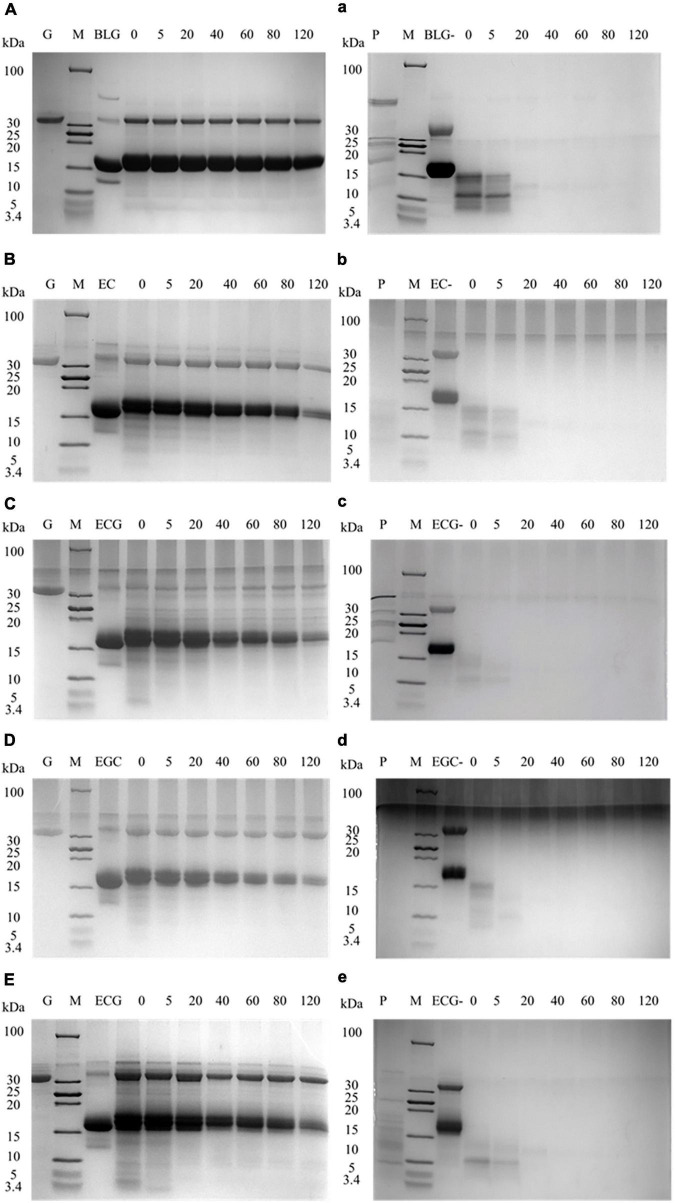
Tricine-SDS-PAGE protein profiles of BLG-catechin complexes digested in simulated gastric fluid and gastrointestinal fluid of adult. **(A–E)** Tricine-SDS-PAGE protein profiles of BLG-catechin complexes digested in simulated gastric of adult. **(a–e)** Tricine-SDS-PAGE protein profiles of BLG-catechin complexes digested in the intestinal fluid of adult. G: pepsin; P: trypsin; M: marker; lane 3: BLG and its complexes (BLG represents the product of gastric digestion for 60 min); lane 4–7: digestion of 0, 5, 20, 40, 60, 80, and 120 min.

Tricine-SDS-PAGE was used to detect the digestion changes in the process of BLG and its covalent complexes. Figures 3A–E represented the gastric digestion diagram of BLG and BLG-catechin complexes. It was found that the molecular weight of BLG hardly changed during gastric digestion. When the catechins were bond, the BLG-ECG, BLG-EGC, and BLG-EGCG were significantly degraded to small peptides or free amino acids after stomach digestion for 40 min, and the degree of degradation increased with gastric digestion time, indicating that catechin can promote the exposure of the BLG cutting site by pepsin. Figures 3a–e represented the intestinal digestibility of BLG and BLG-catechin complexes. The result showed that the final products of gastric digestion were significantly degraded by trypsin. The electrophoresis pattern showed that compared with the untreated BLG, the BLG-catechin complexes was more easily digested by the intestine *in vitro*. Especially, almost no small molecular fragments could be observed after 5 min intestinal digestion of BLG-ECG and BLG-EGC, indicating that the protein was completely degraded. It was found that no bands of BLG and its covalent complexes were observed after simulate gastrointestinal digestion *in vitro*, and all proteins were almost completely digested after 20 min of intestinal digestion. Based on the overall static *in vitro* digestion, it was found that BLG was a relatively resistant protein to gastric digestion, and the addition of all four catechins could promote the gastrointestinal digestion of BLG, especially ECG and EGC.

### Evaluation of transport capacity of digestive products

#### Membrane integrity of two cell models

**FIGURE 4:**
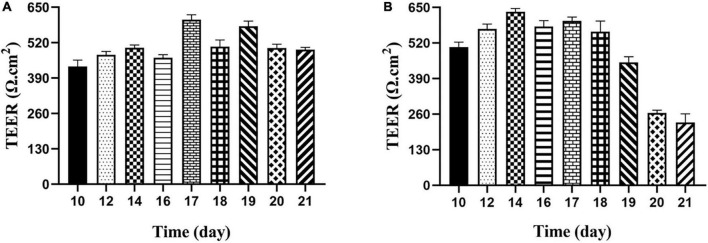
Transepithelial electrical resistance of Caco-2 monolayer model and M cell model. **(A)** Transepithelial electrical resistance of Caco-2 monolayer model; **(B)** transepithelial electrical resistance of M cell model.

It is an important index to evaluate the integrity of cell membrane to measure the resistance value of Transwell plate with resistance meter. According to the previous reports, when trans-epithelial electrical resistance (TEER) was ≥400 Ω⋅cm^2^, it would be selected to indicate membrane integrity (27). The results showed the membrane resistance value on different days, as shown in Figure 4. The resistance value of the Caco-2 monolayer cell model was >400 Ω⋅cm^2^ from the 10th day, and finally reached 493.92 Ω⋅cm^2^ on the 21st day, which increased first and then decreased and became stable with the increase of time, indicating that the structure of monolayer cell membrane was integrated. For the M cell model, as shown from Figure 4B, the resistance value of the co-culture model decreased significantly from the 19th day, and then decreased to about half of the previous resistance value on the 20th day, finally remained stable on the 21st day. When compared with previous reports, the decrease in cell membrane resistance in the M cell model was mainly due to the differentiation of pocket-like M cells with thin cytoplasmic layer, so its resistance value was lower than that of Caco-2 cell membrane (28).

In addition, the permeability of the cell membrane was also an important index to evaluate the cell membrane’ integrity. As a substance with strong yellow-green fluorescence and stable biochemical shape, sodium luciferin has poor permeability, so it is often used to evaluate the permeability of the cell membrane. The standard of membrane permeability was usually determined by the transfer volume of sodium fluorescein <4.8 μg/h⋅cm^2^. According to calculation, the transfer amount of luciferin sodium by Caco-2 and M cell model were 1.621 ± 0.0067 μg/h⋅cm^2^ and 2.918 ± 0.0055 μg/h⋅cm^2^, respectively. It showed clearly that both models were relatively dense and complete. The transit mount of M cell model was twice that of Caco-2 monolayer membranes due to different cells structure, or mutual exclusion between cells, which caused the chimeric ability was abate, the close connectivity between cells was poor.

#### Transport of digestible products in two cell model

**FIGURE 5:**
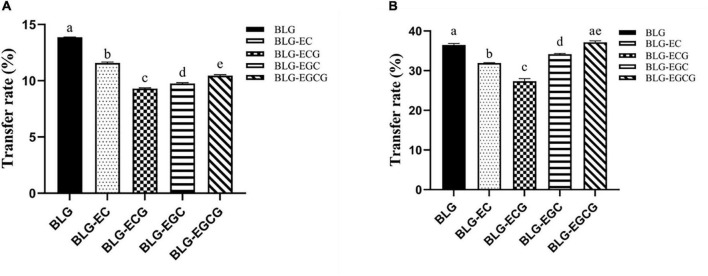
Transport efficiency of digestion of Caco-2 monolayer model and M cell model. **(A)** Caco-2 monolayer model; **(B)** M cell model. Different superscript letters denote significant differences (*p* < 0.05).

The transport rates of the digestible products of digestion of BLG and BLG-catechin complexes in the Caco-2 monolayer cell model and M cell model were shown in Figure 5. In both models, it could be found that the transport efficiency of untreated BLG was the highest, and the transport efficiency of the BLG-catechin complexes was reduced, and the corresponding complex products reflected in the two models were almost the same. Although the addition of catechin can promote the digestion of BLG, catechin may exist in free form after gastrointestinal digestion (29). Due to its small molecular weight and hydrophilicity, small catechin transport substances through the cellular bypass pathway, leading to the filling of the intercellular space and the protection of the intestinal mucosal barrier, thus making it difficult for BLG to pass.

By comparing Figures 5A,B, it can be found that in the M cell model, the transport rates of various digestive products are greatly increased, and the transport rates of all digestive products are basically more than twice that of the Caco-2 monolayer cell model. These results indicated that M cells were successfully differentiated in the co-culture model, and M cells had the ability to transport antigen in the intestinal lumen.

## Discussion

The interactions between polyphenols and proteins were mainly divided into covalent and non-covalent cross-linking. The non-covalent cross-linking products are unstable, easy to decompose and have poor reproducibility due to its mild reaction conditions and weak force. There are three main ways of covalent crosslinking: enzymatic crosslinking, free radical induced crosslinking and alkali crosslinking (30). Among them, the biological enzyme method has specific requirements for the enzyme, and considering the enzyme itself as a high molecular protein has certain allergenicity (31), alkali crosslinking is easy to produce severe Maillard reaction, which has an impact on the protein color, so the free radical induction method with mild reaction and stable product was chosen in our study. The degree of cross-linking reaction between catechins and proteins is related to the structure of catechins. The structure of catechins is mainly composed of a pyran ring and a benzene ring containing substituents. According to whether the third substituent on the pyran ring is gallic ester, catechins can be divided into ester catechins (ECG, EGCG) and non-ester catechins (EC, EGC). In addition, catechins can be divided into dihydroxy catechins (EC, ECG) and trihydroxy catechins (EGC, EGCG) based on the number of phenolic hydroxyl groups in the benzene ring of catechins. It has been reported that the number of binding sites between proteins and catechins is proportional to the number of gallic acid groups contained in catechins (23). In this study, we got the same result. The more the number of polyphenols hydroxyl, the stronger the binding ability with proteins. In the free radical induced reaction, the ortho and para-position of the hydroxyl group on the aromatic ring are vulnerable to free radical attack and react with the protein to form covalent bonds (32).

All the four catechins could cross link with BLG to form complex, and the introduction of catechin had a certain effect on the structural changes of protein. In general, hydrogen bond was the main force to maintain the stability of the secondary structure. While the secondary structure of BLG cross-linked by catechins was not changed a lot, which is consistent with the study of Lu Yuqin (17). We suggested the main molecular force between catechins and BLG is not hydrogen bond. The amino side chain of binding protein was also changed, so it is speculated that a stronger covalent bond is formed between catechins and protein. Therefore, the space structure and the surface hydrophobicity of compounds altered greatly mainly manifested in our study. As we know that increase of ultraviolet absorption spectrum showed that intramolecular chromophore like tryptophan, tyrosine, and phenylalanine exposure (25), peak intensities of BLG bond with catechins raised, which caused the protein surface hydrophobicity, speculated that the effect of catechins and BLG site may reside in a protein on the surface of exposed hydrophobic parts.

The digestion ability of the crosslinked products was related to many factors, such as amino acid composition and sequence, pH-value, temperature, and ionic strength. At present, the construction of intestinal epithelial transport model is mainly focused on cell model *in vitro*. Human colon cancer cells (Caco-2) are similar to human differentiated intestinal epithelial cells, and the cell polarity, tight connection between cells and pinocytosis function are also close to human intestinal epithelial cells. Therefore, Caco-2 is often used to simulate the intestinal transport mechanism of food proteins *in vivo* (33, 34). However, there are also some shortcomings in the transport study of Caco-2 cells: Firstly, Overexpression of tight junction protein leads to high resistance value; Secondly, there is no mucus layer on the tip of Caco-2, which limits the study of protein-mucus interaction. In addition, in terms of the structure of intestinal epithelium, small intestinal epithelial cells consists of intestinal epithelial cells, M cells, goblet cells and part of by cells (35), which M cells can be used as antigen-presenting cell mediated antigen and lumen live bacteria through the epithelial layer to the below immune cells and lead to local or systemic immune response. Therefore, our study compared transport capacity and intestinal epithelial cells between Caco-2 and M cell model. Induction and differentiation of M cells is the perquisite for the construction of M cell model. Gullberg et al. (36) successfully induced differentiation of M-like cells by co-culture mouse PP junction cells and Caco-2 cells for the first time. Later, the researchers (34) found that compared with mouse PP junction cells and Caco-2 cells, Raji B-cells could also differentiate into M cells by co-culture. Moreover, Raji B-cells and Caco-2 cells were used as human cancer cell lines with higher homology and easier operation.

In our study, Caco-2 monolayer cell model and M cell model were simultaneously established as intestinal transport models, using the same number of homologous cells and simultaneously constructed to prevent differences in environment and cell sources. The difference between the two models was that Raji B-cells were added into the monolayer of Caco-2 on day 15 to construct M cell model, and m-like cells were successfully differentiated on day 21. The shape of M cells is pocket-like, and their tight connectivity decreases, resulting in low resistance value. Therefore, the decrease of resistance value can be used as an important indicator for differentiation of Caco-2 cells into M-like cells (37, 38). The M cell model was established in our study under such conditions, and its resistance value decreased to about half of the previous resistance value, which is consistent with previous reports (39). Through transport experiments, it was found that the permeability of the M cell model was low, and the transport efficiency of digestive products in the co-culture model was much higher than that in the Caco-2 monolayer cell model, indicating that M cells had a strong ability to transport antigens. The composition of M cell model is more consistent with the physiological state of human small intestine, so it is more suitable for the study of intestinal transport and absorption mechanism. In this experiment, low transport efficiency indicates better density of the cell model, and the cell model is the construction of the intestinal barrier model. Therefore, low transport efficiency can indicate a healthier intestinal barrier. Similarly, Akbari et al. (40) found that Galacto-oligosaccharide (GOS) modulated the deoxynivalenol-induced decrease in TEER in a concentration-dependent manner, GOSs stimulate the tight junction assembly and in turn mitigate the deleterious effects of deoxynivalenol on the intestinal barrier of Caco-2 cells.

In this study, it was found that the transintestinal transport capacity of the digested products was decreased after the addition of catechin, suggesting that catechin may have a protective effect on the intestinal barrier. Due to its special physiological activity, catechins have been reported to protect and repair the intestinal barrier to some extent. Intestinal function is mainly affected by inflammatory cytokines (41), reactive oxygen species (42) and pathogenic bacteria (43). These mediators not only change the expression of intestinal tight junction proteins, but also affect cytoskeletal binding through activation/inactivation of intracellular signals. Therefore, it is very important to seek a repair method to improve intestinal barrier function injury. Studies have found the regulatory effect of flavonoids, especially catechins, in intestinal tight junction function. Cremonini et al. (44) investigated the effect of EC as a dietary supplement on intestinal barrier in mice fed a high-fat diet, and found that EC inhibited high-lipid-induced ileal NOX1/NOX4 overexpression, protein oxidation, and activation of NF-κB and ERK1/2 pathways, thus protecting against high-lipid-induced increased intestinal permeability. Similarly, Li et al. (45) found that EC can promote intestinal damage after nuclear radiation by regulating oxidative stress response and activating Nrf2 and Wnt/β-catenin signaling pathways to protect the intestinal tract. It has been reported that EGCG pre-treatment can effectively prevent the increase of epithelial cell permeability induced by IFN-γ and IL-4 (46). In addition, catechins can improve the intestinal microbial environment, thereby reducing the intestinal barrier in obese mice (47). In conclusion, catechins play an important role in intestinal barrier protection.

In this paper, it is the first time to explore the potential mechanisms of free radical induced crosslinking between BLG and catechins in green tea both with the structural changes, digestion ability of crosslinked product, furthermore, including the transmembrane transport capacity of digestive crosslinked products. Our results showed that catechins are covalently bound to BLG by C-S and C-N bonds and their binding content was EGCG>EGC>ECG>EC in BLG-polyphenol conjugates. Catechins could change the secondary structure of BLG, decrease the α-helicity, increase the irregular coilings, and lead to the loose spatial structure of the protein. Moreover, catechins could enhance the digested ability of BLG and protect the intestinal barrier *via* the two transintestinal epithelial cell models. Therefore, we deduce that radical induced crosslinking between BLG and catechins in green tea may promote the bioavailability of milk protein.

## Data availability statement

The original contributions presented in this study are included in the article/supplementary material, further inquiries can be directed to the corresponding author.

## Author contributions

XL designed the central ideal of the study. YD performed the research, analyzed the data, and wrote the manuscript. RY and YY performed the research. YW, XM, AY, ZW, LS, and HC developed the idea for the study. All authors contributed to the article and approved the submitted version.

## Footnotes

1
http://dichroweb.cryst.bbk.ac.uk/html/home.shtml
